# Analysis of the miRNA–mRNA–lncRNA networks in ER+ and ER− breast cancer cell lines

**DOI:** 10.1111/jcmm.12681

**Published:** 2015-09-28

**Authors:** Qian Wu, Li Guo, Fei Jiang, Lei Li, Zhong Li, Feng Chen

**Affiliations:** ^1^ The State Key Laboratory of Reproductive MedicineNanjing Medical UniversityNanjingChina; ^2^ Department of Hygienic Analysis and DetectionSchool of Public HealthNanjing Medical UniversityNanjingChina; ^3^ Department of Enviromental Health SciencesBloomburg School of Public HealthJohns Hopkins UniversityBaltimoreMDUSA; ^4^ Department of Epidemiology and Biostatistics and Ministry of Education Key Lab for Modern ToxicologySchool of Public HealthNanjing Medical UniversityNanjingChina; ^5^ Department of Nutrition and Food Hygiene and Ministry of Education Key Lab for Modern ToxicologySchool of Public HealthNanjing Medical UniversityNanjingChina

**Keywords:** oestrogen receptor, mRNA, miRNA, lncRNA, networks

## Abstract

Recently, rapid advances in bioinformatics analysis have expanded our understanding of the transcriptome to a genome‐wide level. miRNA–mRNA–lncRNA interactions have been shown to play critical regulatory role in cancer biology. In this study, we discussed the use of an integrated systematic approach to explore new facets of the oestrogen receptor (ER)‐regulated transcriptome. The identification of RNAs that are related to the expression status of the ER may be useful in clinical therapy and prognosis. We used a network modelling strategy. First, microarray expression profiling of mRNA, lncRNA and miRNA was performed in MCF‐7 (ER‐positive) and MDA‐MB‐231 cells (ER‐ negative). A co‐expression network was then built using co‐expression relationships of the differentially expressed mRNAs and lncRNAs. Finally, the selected miRNA–mRNA network was added to the network. The key miRNA–mRNA–lncRNA interaction can be inferred from the network. The mRNA and non‐coding RNA expression profiles of the cells with different ER phenotypes were distinct. Among the aberrantly expressed miRNAs, the expression levels of miR‐19a‐3p, miR‐19b‐3p and miR‐130a‐3p were much lower in the MCF‐7 cells, whereas that of miR‐148b‐3p was much higher. In a cluster of miR‐17‐92, the expression levels of six of seven miRNAs were lower in the MCF‐7 cells, in addition to miR‐20b in the miR‐106a‐363 cluster. However, the levels of all the miRNAs in the miR‐106a‐25 cluster were higher in the MCF‐7 cells. In the co‐expression networking, CD74 and FMNL2 gene which is involved in the immune response and metastasis, respectively, had a stronger correlation with ER. Among the aberrantly expressed lncRNAs, lncRNA‐DLEU1 was highly expressed in the MCF‐7 cells. A statistical analysis revealed that there was a co‐expression relationship between ESR1 and lncRNA‐DLEU1. In addition, miR‐19a and lncRNA‐DLEU1 are both located on the human chromosome 13q. We speculate that miR‐19a might be co‐expressed with lncRNA‐DLEU1 to co‐regulate the expression of ESR1, which influences the occurrence and development of breast cancer cells with different levels of ER expression. Our findings reveal that the status of ER is mainly due to the differences in the mRNA and ncRNA profile between the breast cancer cell lines, and highlight the importance of studying the miRNA–mRNA–lncRNA interactions to completely illustrate the intricate transcriptome.

## Background

A non‐coding RNA (ncRNA) is a functional RNA molecule that is not translated into a protein (http://en.wikipedia.org/wiki/Non-coding_RNA), and ncRNAs include a diverse of subclasses that are organized based on their size, structure, function and conservation. A well characterized class of ncRNAs is microRNAs (miRNAs), small ncRNAs of approximately 22 nucleotides (nt) in length that are processed from larger precursors. Most of these miRNAs play important roles in a wide range of physiological and pathological processes, including cell differentiation, cell proliferation, development and apoptosis 1, 2. Mature miRNAs are incorporated into the RNA‐induced silencing complex to mediate the transcriptional or post‐transcriptional regulation of gene expression by binding to the 3′‐untranslated regions (3′‐UTR) of the target mRNA 3, 4. Recently, another class of ncRNAs, the long noncoding RNAs (lncRNAs) have gained increased attention 5, 6. Unlike the miRNAs, lncRNAs are longer, more than 200nt in length and usually have complex and diverse sequences. Generally, based on the position of the lncRNA relative to the neighbouring protein‐coding genes in the genome, lncRNAs can be divided into five categories, namely: sense, antisense, bidirectional, intronic and intergenic 7. Although lncRNAs have been investigated less often than miRNAs, a number of lncRNAs have been functionally characterized, such as HOTAIR 8, 9, Xist 10 and H19 11, 12, 13. Recent reports have shown that lncRNAs are involved in the regulatory roles in diverse processes, such as imprinting, X‐inactivation and development. In addition, lncRNAs are also known to be associated with the pathogenesis of different kinds of diseases including cancers 6, 14, 15. Although much of the focus in ncRNA research is on directly understanding the ncRNA‐mediated regulation of mRNA, it has been suggested that ncRNAs and mRNA could form a well‐regulated interacting network 16. There have been reports that suggested examples of such regulations, *i.e*. miRNA–miRNA, miRNA–mRNA, lncRNA–mRNA and miRNA–lncRNA interactions 17, 18, 19. The entire paradigm of the ncRNA regulatory layer remains incompletely explored. Therefore, additional research is required to investigate the relationships among miRNAs, lncRNAs and mRNAs in the biological process of cancer.

Nearly 70% of breast cancers (BCs) over express the oestrogen receptor (ER) 20. The up‐regulation of the ER during the early stages of tumourigenesis has been identified as an important factor in stimulating the proliferation of mammary cells leading to tumour development 21. The presence of the ER is the main indicator for antihormonal therapy 22. The molecular subtypes of ER in human BCs are characterized by different responses to the therapy, differential course and prognosis 23. In most cases, ER‐positive (ER+) BCs present a better clinical prognosis than those that are ER negative. The ultimate step in the progression of BC is metastasis. Within the BC subtypes, those characterized by the lack of expression of hormone receptors exhibit dismal survival rates due to the highly aggressive and metastatic behaviour of these BCs 24, 25. Moreover, the absence of novel therapies capable of specifically targeting this very aggressive subtype reflects, in part, a lack of sufficient knowledge regarding ER‐negative (ER−) BC development and progression 26, 27.

The differences of the ER+ and ER− BC not only relate to their morphology, but also are largely due to the difference in their transcriptional responses, and it is necessary to examine the miRNA–mRNA–lncRNA network in ER+‐and ER− cell lines in order to cover the diversity of breast carcinomas in a more extensive way. In the present study, the lncRNA, miRNA and mRNA expression profiles were compared in the MCF‐7, low metastatic, ER+ BC cell line and the MDA‐MB‐231, highly metastatic, ER− BC cell line using microarray technology to compare interrelated factors that regulate BC progression. An integrated analysis among the three groups of RNA within different genetic networks was used to identify genes and pathways that may be related to ER expression in the MCF‐7 cells. To date, this study is one of the first transcriptome‐wide studies on ncRNA–mRNA interactions between MCF‐7 and MDA‐MB‐231 cell lines. We hope further experimental analyses will reveal new mechanistic insights into the function and regulation of ncRNAs.

## Materials and methods

### Cell culture

Human BC cell lines MCF‐7, and MDA‐MB‐231 were gifts from Prof. Zhong Li, Nanjing Medical University, Nanjing, China. The MCF‐7 and MDA‐MB‐231 cells were maintained in DMEM/L‐15 medium (Gibco, BRL, Gaithersburg, MD, USA) supplemented with 100 μg/ml streptomycin (Sigma‐Aldrich, St. Louis, MO, USA), 100 U/ml penicillin (Sigma‐Aldrich) and 10% fetal calf serum (Sijiqing, Hangzhou, China) in humidified air with or without 5% CO_2_ at 37°C. Cells in the logarithmic phase of growth were used for microarray analyses.

### Subjects

Cancerous breast tissue was obtained from 31 BC patients with ER+ BC, and 27 with ER− BC, which were collected from Jiangsu Provincial People's Hospital. Written informed consent was obtained from the participants for the use of tissue samples in this study. This study was approved by the Nanjing Medical University Clinical Research Ethics Committee, Nanjing, China. No patients received chemotherapy or radiotherapy before tissue samples were collected.

### miRNA microarray

Microarray profiling for miRNA was performed with Agilent Human miRNA 8 × 60k v.18.0 arrays (Agilent Technologies, Santa Clara, CA,USA) by CapitalBio (Capital‐Bio Corp., Beijing, China). Briefly, 10μ g of total RNA was purified by using mirVana miRNA isolation kit (Ambion, Austin, TX, USA) to enrich the small RNA fraction. The purified RNA was labelled with Cy3 and hybridization was carried out on the 8 × 60 K Agilent miRNA array, corresponding to 1887 human miRNA genes, with the Agilent miRNA Complete Labeling and Hyb Kit.

The miRNA array data were analysed for data summarization, normalization and quality control by using the GeneSpring software v.11.5 (Agilent Technologies, Inc). To select the differentially expressed genes, we used threshold values of ≥2 and ≤−2‐fold change and a Benjamini–Hochberg corrected *P* value of 0.05. The data were log_2_ transformed and median centred by genes using the Adjust Data function of CLUSTER 3.0 software and then further analysed with hierarchical clustering with average linkage. Finally, we performed tree visualization by using Java TreeView (Stanford University School of Medicine, Stanford, CA, USA). miRNA microarray was performed in triplicates for each cell line.

### lncRNA+mRNA microarray

Briefly, isolates from MCF‐7/MDA‐MB‐ 231 cells were used to synthesize double‐stranded complementary DNA (cDNA). Double‐stranded cDNA was labelled and hybridized to the 4 × 180 K Agilent human lncRNA+mRNA Array v2.0. The array contains about 39,000 human lncRNAs and 32,000 human mRNAs. These lncRNA and mRNA target sequences were merged from the existing databases, such as RefSeq and Ensembl (Table S1). After hybridization and washing, the processed slides were scanned with the Agilent G2565CA Microarray Scanner. The lncRNA+mRNA array data were analysed for data summarization, normalization and quality control by using the GeneSpring software v.11.5 (Agilent Technologies, Inc). To select the differentially expressed genes, we used threshold values of ≥2 and ≤−2‐fold change and a Benjamini–Hochberg corrected *P* value of 0.05. The data were log_2_ transformed and median centred by genes using the Adjust Data function of CLUSTER 3.0 software (University of Tokyo, Human Genome Center, Tokyo, Japan) then further analysed with hierarchical clustering with average linkage. Finally, we performed tree visualization by using Java TreeView (Stanford University School of Medicine). The lncRNA+mRNA microarray was performed in triplicates for each cell line.

### Analysis of lncRNA quantification

Real‐time RT‐PCR was used to verify the differential expression of selected genes that were detected with the lncRNA expression microarray. The cDNA was synthesized using PrimeScript^™^RT Master Mix (Perfect Real Time; TaKaRa Biotechnology, Dalian, China). Each real‐time PCR reaction (in 20 μl) contained 2× SYBR Premix Ex Taq (Tli RnaseH Plus; TaKaRa), 0.2 μM primers and 2 μl of cDNA. The cycling conditions consisted of an initial, single cycle of 30 sec. at 95°C, followed by 40 cycles at 95°C, 5 sec. and 60°C, 31 sec. PCR amplification was performed in three duplicates for each sample. Gene expression levels were quantified relative to the expression of glyceraldehyde‐3‐phosphate dehydrogenase (GAPDH) using an optimized comparative Ct (2^−∆Ct^) value method. Two‐tailed Student's *t*‐test was performed.

### miRNA quantification by real‐time PCR

MicroRNA quantification was performed by SYBR green quantitative RT‐PCR (qRT‐PCR) assay. Briefly, the total RNA of cells and tissue was extracted using a miRNeasy Mini Kit (Qiagen, Hilden, Germany). The miRNA was reverse transcribed with SYBR PrimeScript miRNA RT‐PCR Kit (TaKaRa). Realtime qPCR was performed with SYBR Premix Ex Taq II (Perfect Real Time; TaKaRa) with the manufacture‐provided universal primer and the miRNA‐specific forward primers designed by RiboBio (Guanzhou, China) in ABI PRISM 7300 real‐time PCR system (Applied Biosystems, Foster City, CA, USA). Each reaction was performed in a final volume of 20 μl containing 2 μl of cDNA, 10 μl of 2× SYBR Premix Ex Taq II, 1.8 μl of PCR forward primer, and 0.8 μl of Uni‐miR qPCR primer. The amplication profile was as follows: denaturation at 95°C for 30 sec., followed by 40 cycles at 95°C for 5 sec. and 60°C for 31 sec. Each sample was run in duplicates for analysis. The relative amount of miRNAs was normalized against U6 snRNA, and the fold change for each miRNA was calculated by the 2^−∆Ct^ method. The significance of miRNA levels was determined by Mann–Whitney. All of the *P* values were two sided, and a value less than 0.05 was considered statistically significant. All of the statistical calculations were performed with the SPSS software (SPSS Inc., Chicago, IL, USA) (v. 16.0) and GraphPad Prism 5 Demo software (GraphPad software, San Diego, CA, USA).

### Bioinformatics analysis

**Figure 1:**
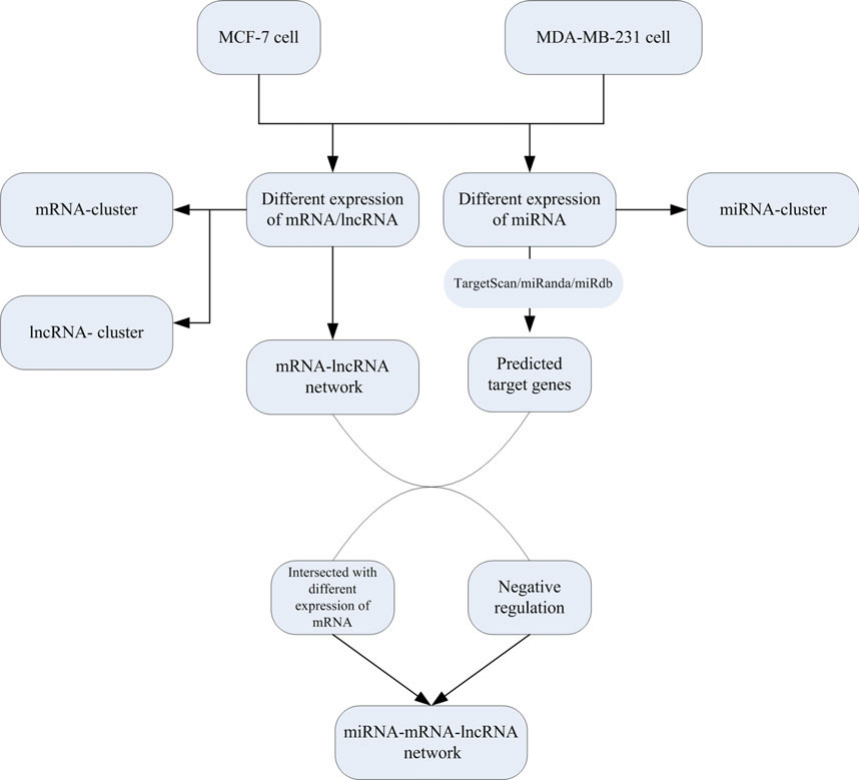
miRNA–mRNA–lncRNA network analysis flow chart.

Predicted targets of miRNAs differentially expressed in this study were determined using mRBase targets (http://mirdb.org/miRDB/, http://www.targetscan.org/and http://www.microrna.org/microrna/). In addition, we used the Gene Ontology database (http://www.geneontology.org) to perform gene ontology (GO) analysis on the target genes. After the analyses for significance and false discovery rate (FDR), GO terms were selected from the significantly enriched gene sets (FDR <0.05). Pathway analysis was used to identify significant pathways for the differentially expressed genes according to the Kyoto Encyclopedia of Genes and Genomes (KEGG) 28. The significant pathways were selected by Fisher's exact and chi‐square tests. Then we constructed gene co‐expression network to identify gene interactions. The gene co‐expression network was built according to the normalized signal intensity of specific expressed genes. We calculated the Pearson correlation coefficient between two genes. Only the strong correlations (0.99 or greater) were selected to construct the network. Figure 1 depicts a flowchart for bioinformatics analysis.

### Luciferase reporter assay

Two pmiR‐RB‐REPORT^™^‐ESR1‐3′‐UTR recombinants, containing 3′‐UTR fragments of the ESR1 gene with or without the putative binding site for miR‐19a, b, were constructed. DNA fragments with and without the miR‐19 target sequence was amplified *via* PCR using human DNA as the template. The product was cloned into the SgfI/NotI site of the pmiR‐RB‐REPORT^™^ vector (RiboBio) to generate the vector pmiR‐RB‐REPORT^™^‐ESR1/3′UTR. The ligated vector was amplified in DH5α. Antisense and sense primers of the vector were used to screen the clones, which were further verified by sequencing. The resulting luciferase UTR‐reporter vectors (100 ng/well) and 50 nM of miR‐19a,b mimic were cotransfected into 293 T cells using Lipofectamine^™^ 2000 reagent according to the manufacturer's protocol (Invitrogen, Carlsbad, CA, USA). Forty‐eight hours after transfection, luciferase activity assays were performed with Dual ‐Glo Luciferase Assay System (Promega, Madison, WI, USA) following the manufacturer's instructions.

### Transfection

The three siRNAs specific for DLEU1 and miR‐19a,b mimics used in this study were purchased from RiboBio, and were transfected to MCF‐7 cells at a working concentration of 50 or 100 nM using Lipofectamine 2000 (Invitrogen) at 24 hrs following seeding cells. Control oligonucleotides were used as controls (RiboBio). (The sequences siRNAs specific for DLEU1 are shown in Table S2). The transfected cells were used for the analysis of ESR1 expression.

### Western blot analysis

MCF‐7 cells were collected and analysed using Western blot to assess ESR1 expression. Antibody against ESR1 was purchased from Thermo, Waltham, MA, USA. The ESR1 antibody was diluted to 1:500 working concentration. Glyceraldehyde‐3‐phosphate dehydrogenase was used as a loading control on the same membrane.

### Bisulphite genomic sequencing

The DNA methylation status of the lncRNA‐DLEU1 promoter was assayed by sodium bisulphite methylation sequencing. Genomic DNA (200 ng) from MCF‐7 and MDA‐MB‐231 cells was modified with sodium bisulphate by using the EZ Methylation Modification Kit (Zymo Research, Orange, CA, USA) and then amplified by PCR using Go Taq mix (Promega). *In silico* analyses and detailed databases searches were used to predict the 5′‐CGI(s) in lncRNA‐DLEU1 gene. PCR primers were designed to amplify a CpG‐rich region spanning from −1500 to −1000 bp from the transcription start site, which contains 32 CpG sites. Bisulphite primer sequences were 5′‐GAGTTGTGGAGTAAGAATTGATAGAAATTATTAGTTA‐3′ for forward and 5′‐CAAACCC(T)GAAATCATAAATCCCTC‐3′ for reverse. Amplicons were subcloned into the pCR2.1 vector using the Original TA Cloning Kit (Invitrogen); minimum of ten clones were picked and sequenced (Generay, Shanghai, China). Percent methylation was calculated using the following formula: Number of methylated CpGs×100/total number of CpGs assessed. TRANSFAC@7.0 (Qiagen, Hilden, Germany) was used to identify transcription factor binding sites within the lncRNA‐DLEU1 promoter region.

### Statistics

Data from independent triplicate (*n* = 3) experiments were collected and statistical significance between MCF‐7 and MDA‐MB‐231 cells were determined by one‐way anova and Tukey–Krammer Multiple Comparison test using GraphPad Prism V6.0 software (GraphPad). A *P* < 0.05 was considered statistically significant.

## Results and discussion

### Microarray analysis of MCF‐7 and MDA‐MB‐231 cell lines

**Figure 2:**
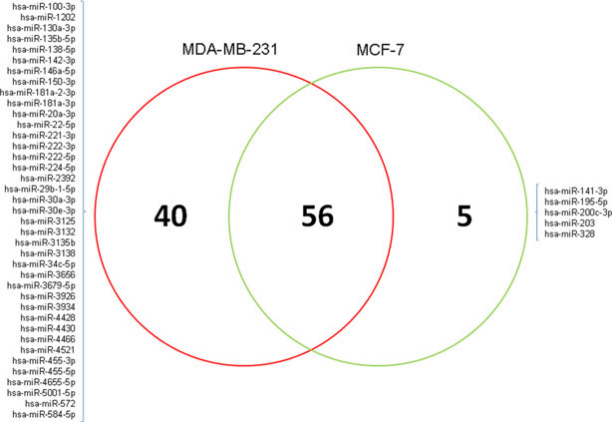
Venn diagram analysis of miRNAs differentially expressed between MCF‐7 and MDA‐MB‐231 cells. The differential miRNA expression between MCF‐7 and MDA‐MB‐231 cells is represented graphically in a Venn diagram. MCF‐7 had 61 and MDA‐MB‐231 had 96 significant miRNAs, and 56 miRNAs intersecting between the circles were common.

**Figure 3:**
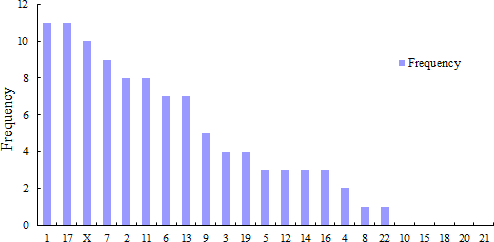
The distribution of miRNA in human chromosomes.

To investigate differences in the expression of miRNAs between MCF‐7 and MDA‐MB‐231 cell lines, miRNA expression profiles were assessed using the Agilent miRNA arrays. The results showed that in MCF‐7 cells, 36 miRNAs were highly expressed and the expression of 65 was diminished compared with that of MDA‐MB‐231 cell line (fold change >2.0, *P* < 0.05, Table S3 and S4, Figure S1). In these differentially expressed miRNAs, five miRNAs were only detected in MCF‐7, 40 only in MDA‐MB‐231 cells and 56 in both cell lines (Fig. 2). The distribution of these miRNAs among the human chromosomes is depicted in Figure 3.

Chromosome 1, 17, X, 7 and 2 have high number of miRNA genes, almost up to 50%. In humans, cancer associated miRNAs are mostly located in Chromosome 19, X, 14 and 1 29.

In the current study, the level of miR‐141 and miR‐200c was higher in MCF‐7 relative to MDA‐MB‐231 cells. miR‐141 and miR‐200c belongs to miR‐200 family. There are growing evidence to suggest that miR‐141 and miR‐200c plays an essential role in tumour suppression by inhibiting epithelial–mesenchymal transition (EMT) and metastasis by direct targeting of E‐cadherin transcriptional repressors *ZEB1* and *ZEB2* 30, 31, 32.

Hurteau *et al*. 33 combined bioinformatics with quantitative RT‐PCR to reveal that the expression *ZEB1* was inversely proportional to that of miR‐200c. In MCF‐7 cell, miR‐200c was endogenously expressed, but *ZEB1* was absent. In contrast, MDA‐MB‐231 cell lacked detectable miR‐200c, but expressed *ZEB1*. The ectopic expression of miR‐200c in MDA‐MB‐231 cell reduced the level of *ZEB1*, and altered cell morphology. Loss of miR‐200c expression could play a significant role in the initiation of an invasive phenotype, and, equally, miR‐200c overexpression holds potential for its reversal. *ZEB1* can suppress the transcription of miR‐141 and miR‐200c, and forms an miRNA‐mediated feed forward loop that stabilizes EMT and promotes the invasion of cancer cells 34. In addition, the expression level of *ZEB1* in MDA‐MB‐231 cell was really higher than that of MCF‐7 cell in our gene expression results (Table S5).

**Figure 4:**
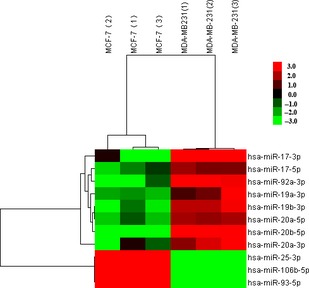
Heat map of differentially expressed miR‐17‐92 cluster and its two paralogues. Compared with MDA‐MB‐231 cell, the expression of miR‐17‐3p, miR‐17‐5p, miR‐92a‐3p, miR‐19a‐3p, miR‐19b‐3p, miR‐20a‐3p, miR‐20b‐5p and miR‐20a‐3p was higher, and that of miR‐25‐3p, miR‐106b‐5p and miR‐93‐5p was lower in MCF‐7.

In the analysis of the differential expression of miRNAs, most of miR‐17‐92 cluster and its two other paralogues, the miR‐106b‐25 and the miR‐106b‐363 clusters, were differentially expressed, such as miR‐17‐5p, miR‐17‐3p, miR‐19a‐3p, miR‐20a, miR‐19b‐3p and miR‐92‐3p, miR‐93‐5p, miR‐25‐3p and miR‐20b (Tables 1 and 2, Fig. 4).

**Table 1:** The differential expression of the miR‐17‐92 cluster and its two paralogues in MCF‐7 and MDA‐MB‐231 cells

miRNA	Fc (fold change)	Regulation
miR‐17‐5p	2.44	Down
miR‐17‐3p	3.60	Down
miR‐19a‐3p	2.62	Down
miR‐20a‐3p	29.43	Down
miR‐20a‐5p	2.58	Down
miR‐19b‐3p	2.86	Down
miR‐92a‐3p	3.35	Down
miR‐20b‐5p	3.66	Down
miR‐106b‐5p	2.58	Up
miR‐93‐5p	2.81	Up
miR‐25‐3p	2.61	Up

**Table 2:** ESR1‐related co‐expression of mRNA/lncRNA in MCF‐7 cells

mRNA	mRNA/lncRNA	Interaction
ESR1	XLOC_002797	−0.999992381
ESR1	uc001ver.2	−0.999989273
ESR1	NR_037174	−0.999980388
ESR1	FMNL2	0.999954187
ESR1	CD74	0.999993841

The miR‐17‐92 cluster, encoding 7 miRNAs, miR‐17‐5p, miR‐17‐3p, miR‐18a, miR‐19a, miR‐20a, miR‐19b‐1 and miR‐92a‐1, is a polycistronic cluster on human chromosome 13(+), whereas the two other paralogues, miR‐106b~25 cluster, encoding miR‐106b‐5p, miR‐93‐5p and miR‐25‐3p and the miR‐106a~363 cluster, encoding miR‐106a, miR‐18b, miR‐20b, miR‐19b‐2, miR‐92‐2 and miR‐363, are located on human chromosomes 7 and X respectively. Overexpression of the miR‐17‐92 cluster is observed in a variety of cancers, including small‐cell lung cancer, colon cancer, neuroblastomas, medulloblastoma and gastric cancer 35, 36, 37, 38. Calvano *et al*. 39 used real‐time RT‐PCR to evaluate the miRNA expression profiles of formalin‐fixed paraffin‐embedded BC tissue. It was found that the expression of miR‐17‐5p, miR‐18a‐5p and miR‐20a‐5 in the triple‐negative tumours (ER−, PR− and HER2−) was higher that of luminal A samples. Li *et al*. demonstrated that down‐regulation of endogenous miR‐17‐5p suppressed the migration and invasion of MDA‐MB‐231 cells by targeting HBP1 and subsequent activation of Wnt/β‐caten 39, 40. These findings suggest that the differences in the expression of miR‐17‐92 cluster will explain the phenotypic differences between these molecular subtypes of tumours.

**Figure 5:**
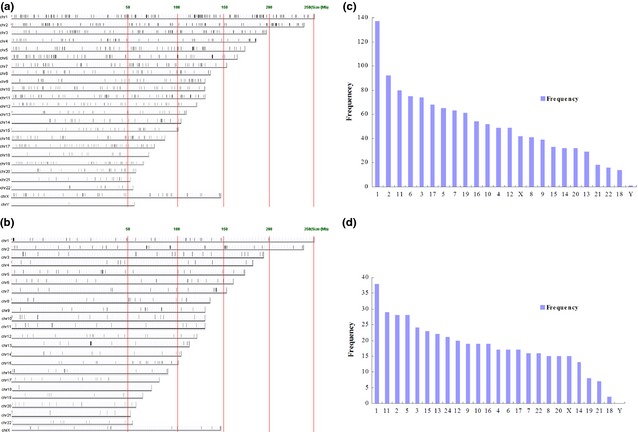
The distribution of differentially expressed mRNAs (**A** and **C**) and lncRNAs (**B** and **D**)in human chromosomes.

Long non‐coding RNA and mRNA profiles were obtained using the 4 × 180K Agilent human lncRNA+mRNA Array v2.0. Hierarchical clustering showed systematic variations in the expression of lncRNA and protein‐coding mRNAs between the two cell lines (Figure S2A and B). Compared with MDA‐MB‐231 cell, 449 lncRNA were differentially expressed in MCF‐7 cell, of which 298 were up‐regulated, and 151 down‐regulated. There were three criteria: (*i*) fold change >2.5 and *P* < 0.05; (*ii*) the lncRNA sequence does not match with protein‐coding region; (*iii*) the lncRNA sequence length is less than 2 kb (Tables S6 and S7). And compared with MDA‐MB‐231 cell, there were 1216 differentially expressed mRNA, of which 558 up‐regulated, and 658 down‐regulated (fold change >5; *P* < 0.001) in MCF‐7 cell (Tables S5 and S8). The distribution of differentially expressed mRNA and lncRNA in human chromosomes was visualized by UCSC genomic browser (Fig. 5 A–D), most of which were localized on chromosomes 1, 2, 3, 5, and 11.

### Microarray‐based GO analysis and pathway analysis

Predicted targets of 101 miRNAs differentially expressed in this study were determined by using three database: mirDB, TargetScan and Miranda. A total of 2066 target mRNAs were predicted, 723 of which, including ESR1, were significantly different between MCF‐7 and MDA‐MB‐231 cells by intersecting with mRNA microarray (Table S9).

**Figure 6:**
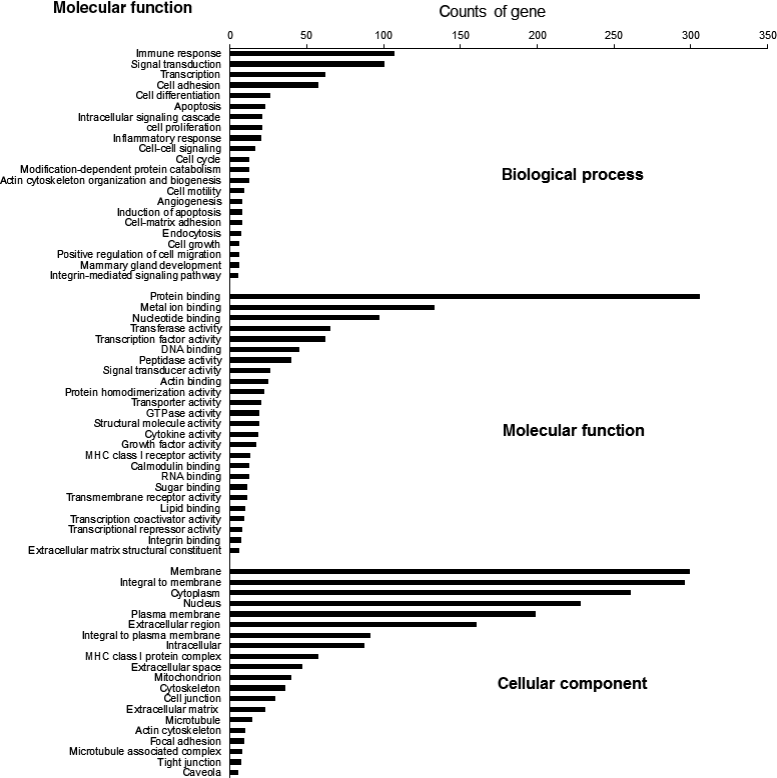
GO term classification of 723 differentially expressed genes. Counts of gene represents the number of genes annotated by gene ontology database to the GO terms, BP: biological process; MF: molecular function and CC: cellular component.

Then, we used Gene Ontology database (http://www.geneontology.org) to perform GO analysis on the target genes. The up‐ and down‐regulated genes were individually analysed. The *P* value and FDR was calculated by Fisher's exact test and multiple comparisons test respectively (*P* < 0.05, Table S10). Around 723 differentially expressed genes were classified according to GO term, including biological process, BP; molecular function, MF and cellular component, CC (Fig. 6). And specific biological process categories were enriched, such as apoptosis, cell migration, cell cycle, cell proliferation, cell adhesion, response to drug, angiogenesis, immune response and EMT.

**Figure 7:**
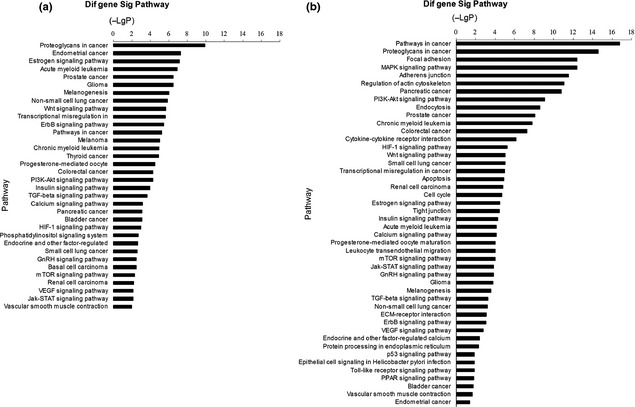
KEGG pathway analysis of differentially expressed gene between MCF‐7 and MDA‐MB‐231 cells. Pathway analysis was used to place differentially expressed genes according to KEGG. Fisher's exact tests were used to identify pathways, and the threshold of significance was defined by the *P* value. X represents the log *P* value. (**A**) Up‐regulated genes mainly participate in oestrogen signalling pathway, ErbB signalling pathway, and Wnt signalling pathway, *etc*. (**B**) Down‐regulated gene in signalling pathway, MAPK signalling pathway, PI3K‐AKT signalling pathway, HIF‐1 signalling pathway, mTOR signalling pathway, TGF‐β signalling pathway, ECM‐receptor interaction, VEGF signalling pathway and p53 signalling pathway, *etc*.

Pathway enrichment analyses were performed based on KEGG pathway analysis using either Chi‐square test or Fisher's exact test. Pathways with more annotations than expected among the differentially expressed genes (*P* < 0.05) were considered significantly enriched. Figure 7 displays pathway analysis of differentially expressed gene in MCF‐7 and MDA‐MB‐231 cells. Up‐regulated genes mainly participate in oestrogen signalling pathway, ErbB signalling pathway and Wnt signalling pathway, *etc*. Many of down‐regulated genes are linked to cell motility‐related mechanisms, a critical step in the promotion of cancer invasion and metastasis, such as, focal adhesion, adherens junction, tight junction, regulation of actin cytoskeleton and control of membrane proteins through endocytosis. Others relate to intracellular signalling pathways, such as MAPK signalling pathway, PI3K‐AKT signalling pathway, HIF‐1 signalling pathway, mTOR signalling pathway, TGF‐β signalling pathway, ECM‐receptor interaction, VEGF signalling pathway and p53 signalling pathway, *etc*.

### miRNA–mRNA–lncRNA interaction

First, we constructed gene co‐expression networks to identify interactions among differentially expressed mRNA and lncRNAs. Gene co‐expression networks were built according to the normalized signal intensity. We then calculated the Pearson correlation coefficients. To make a visual representation, only the strongest correlations (0.99 or greater) were included. In this representation, each gene corresponded to a node and the connection of two genes was represented by an edge, indicating a strong correlation (*i.e*. either positive or negative). A degree was defined as the number of directly linked neighbours. The miRNA–mRNA interaction was integrated into the co‐expression networks according to the negative regulation. The co‐expression networks were drawn using Cytoscape 3.0. In addition, we used two online databases to find out BC‐related genes and oestrogen responsive genes, which were integrated in our network (http://bioinf-data.charite.de/cancerresource/index.php?site=somatic_cancer, http://datam.i2r.a-star.edu.sg/ergdbV2/) 41.

**Figure 8:**
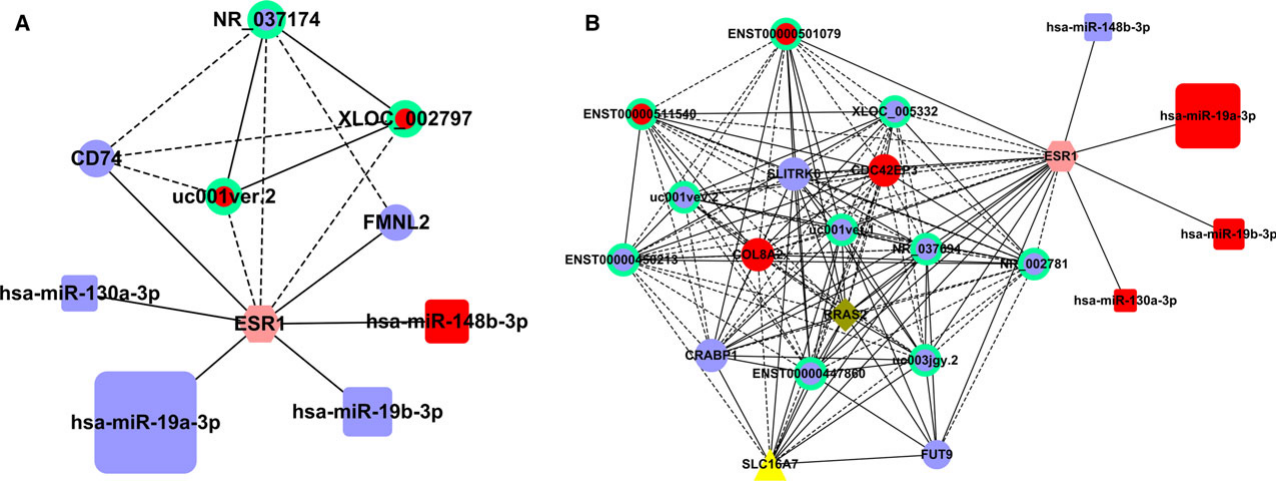
miRNA‐ESR1‐lncRNA network. Figure 8 showed mRNA‐ESR1‐related miRNA–mRNA–lncRNA network in MCF‐7 (**A**) and MDA‐MB‐231 (**B**) cells respectively. The dots represent the co‐expressed genes. The dots in a circle represent the lncRNA co‐expression. The squares represent miRNA expression. All red represents up‐regulated and blue represents down‐regulated. In addition, oestrogen responsive genes and breast cancer‐related genes were added into the network. The yellow dots represent oestrogen responsive genes, the dark green dots represent breast cancer‐related genes, and the pink dots represent the intersection of the two set above. The yellow triangle, the dark green diamond and the pink hexagon represent oestrogen responsive genes, breast cancer‐related genes, and the intersection which were aberrantly expressed in the network respectively (Fig. S1). In mRNA–mRNA, mRNA–lncRNA and lncRNA–lncRNA interaction, solid line represents positive regulation, and the dotted line represents negative regulation.

The structure of the co‐expression networks of MCF‐7 and MDA‐MB‐231 cells were significantly different (Figs S3 and S4), indicating that the co‐expression patterns of lncRNAs and mRNAs in MCF‐7 and MDA‐MB‐231 cells were different. The ERα (ESR1)‐related miRNAs and lncRNAs were then extracted (shown in Figs S5 and S6). Furthermore, we selected pairs (only lncRNA–mRNA) with the strong correlations (>0.9999) to construct the miRNA‐ESR1‐lncRNA network (Fig. 8A and B).

In the MCF‐7 co‐expression network, mRNA‐ESR1 is connected to two mRNAs, including positively regulated genes CD74 and FMNL2 and three lncRNAs, and including the negatively regulated uc001ver.2 (DLEU1) (Table 2). As ESR1‐target gene, miR‐130a‐3p, miR‐19a‐3p, miR‐19b‐3p and miR‐148b‐3p was differently expressed with statistical significance, of which miR‐130a‐3p, miR‐19a‐3p and miR‐19b‐3p had lower expression levels, and miR‐148b‐3p had a higher expression level in MCF‐7 cells.

CD74 is the γ subunit of major histocompatibility complex class II and is an important chaperone protein that regulates antigen presentation for the immune response. Some studies have demonstrated that CD74 protein is overexpressed in human cancers, such as BC, gastric cancer and pancreatic cancer 42, 43, 44. High levels of CD74 expression which might prevent the presentation of tumour antigen to T cells 42. CD74 may be a key factor of proliferating and migrating cells; however, the mechanisms involved in the functions or regulation of CD74 are not fully understood. Another study based on tissue samples suggested that triple negative tumours with Stat1/CD74‐positive are more aggressive and suggested a new target for diagnostics and more targeted therapies for triple‐negative BC 45.

Formins are proteins that govern cell shape, adhesion, cytokinesis and morphogenesis by remodelling actin exerted through the formin homology domains 46. They frequently are deregulated during tumour cell transformation and metastasis 47. Formin‐like 2 (FMNL2) is a novel member of Diaphanous‐related formins group 48. Overexpression of FMNL2 in colorectal cancer tissues and cell lines was found to be associated with invasion and lymphatic metastasis by inducing EMT 49, 50, 51, 52.

### ESR1 mRNA – related miR‐19 and DLEU1

**Figure 9:**
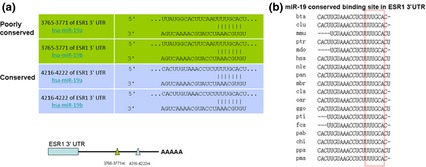
The putative miR‐19a targeted sequence in the ESR1 gene. (**A**) TargetScan predicts two binding sites in ESR1 3′UTR. Because of the perfect homology between the seed regions of miR‐19a and b, both of these microRNAs are able to target the same sequence in the 3′UTR. (**B**) Conservation of miR‐19a and b binding site in ESR1. 3′UTR is shown across several species.

**Figure 10:**
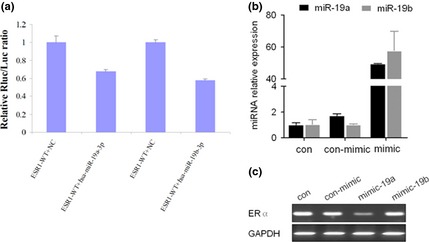
ESR1 is a predicted target of miR‐19. (**A**) Luciferase reporter assays. The luciferase activity of the transfected with the vector containing the ESR1 3′UTR fragment with the binding sequence of miR‐19a and b was inhibited by transfection of miR‐19a and b mimic into 293 T cells. (**B**) Efficient accumulation of miRNAs. MCF‐7 cells were transfected with either scrambled oligo (Ctrl) or miR‐19a and b mimics. The cells were harvested 24 hrs after transfection and total RNA was isolated. (**C**) The expression levels of ESR1 mRNA in miR‐19a mimics‐transfected MCF‐7 cells determined by real‐time RT‐PCR were significantly down‐regulated compared to the control cells.

miR‐19 was predicted by software to bind the 3′UTR of ESR1 at two different sites, A and B (Fig. 9). Site B is highly conserved across several species, whereas site A is poorly conserved. To determine whether miR‐19 targets the ESR1 3′UTR, a luciferase reporter in the pmiR‐RB‐REPORT^™^ vector containing the full‐length ESR1 3′UTR was constructed. Overexpression of miR‐19 statistically significantly inhibited ESR1 3′UTR luciferase activity relative to the scrambled sequence control (Fig. 10A). We concluded that miR‐19 targets ESR1 by binding sites within the 3′UTRs.

**Figure 11:**
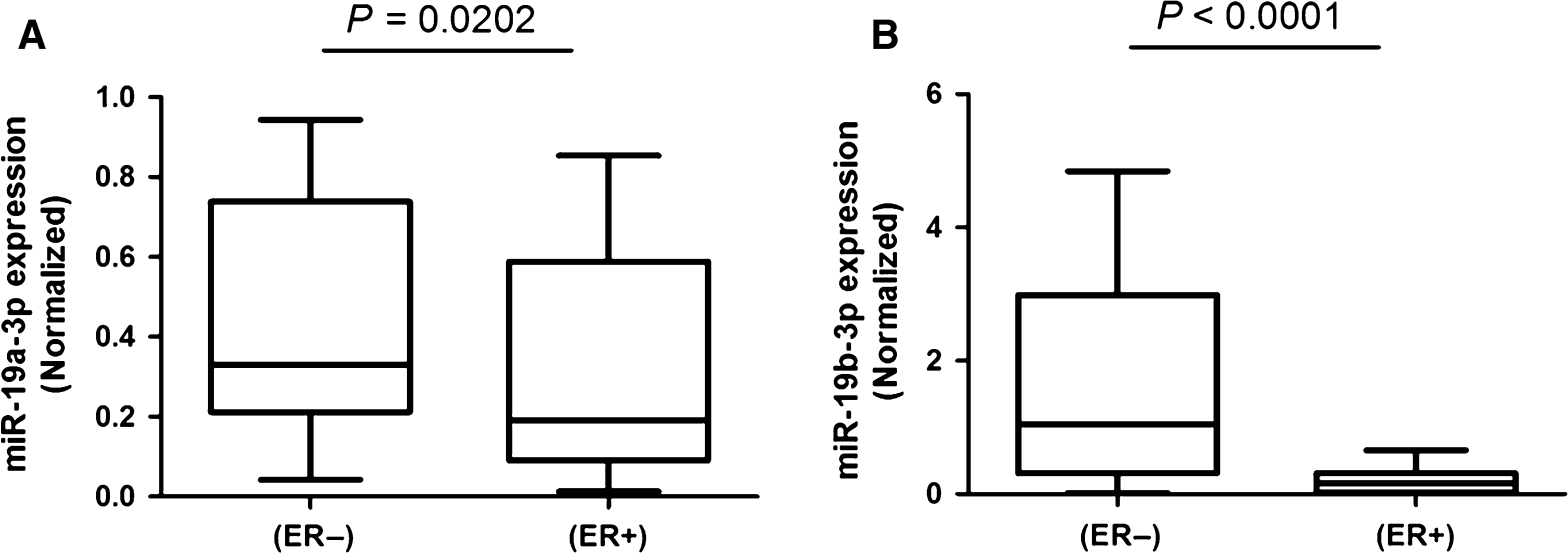
Box and whiskers plot of tissue levels of (**A**) miR‐19a and (**B**) miR‐19b in ER positive (ER+, *n* = 37) and ER negative samples (ER−, *n* = 27). The expression level of miRNAs was normalized to that of RUNB6. Whiskers represent the min to max value. The statistically significant differences were determined by Mann–Whitney test using GraphPad Prism 5 Demo software.

**Figure 12:**
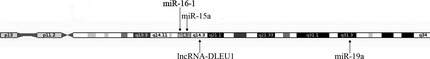
The location of miR‐19a and lncRNA‐DLEU1 on chromosome 13 (www.ensembl.org).

**Figure 13:**
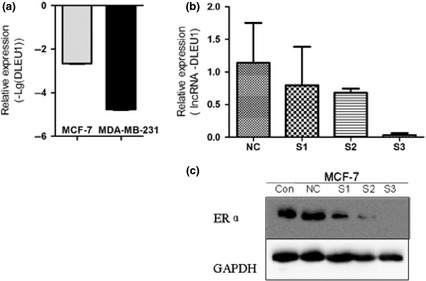
A pos itive correlation between lncRNA‐DLEU1 and ESR1. (**A**) Expression levels of lncRNA‐DLEU1 in MCF‐7 and MDA‐MB‐231 cells. (**B**) The efficiency of the three siRNA candidates was confirmed by qRT‐PCR. (**C**) The protein expression levels of ER in lncRNA‐siRNA‐transfected MCF‐7 cells were down‐regulated compared to those of the control oligonucleotides‐transfected MCF‐7 cells.

To determine the differential effect of miR‐19a‐3p and miR‐19b‐3p on ERa production, MCF7 cells were transfected with these two miRNAs and a scrambled sequence miRNA control. quantitative RT‐PCR showed efficient accumulation of miRNAs (*P* value of difference between the samples and the control <0.05; Fig. 10B). This accumulation was followed, as expected, by a suppression of ERa expression. quantitative RT‐PCR demonstrated that only miR‐19a‐3p substantially reduced expression of ERa mRNA (*P* < 0.05; Fig. 10C). And then we examined the expression of miR‐19 in cancerous tissue sample with different expression pattern of ER. The expression of both miR‐19a and b in BC tissue samples with ER+ were down‐regulated compared to those with ER−, indicating that miR‐19a may negatively regulate the expression of ER (Fig. 11). In co‐expression networks, lncRNA‐DLEU1 was strongly connected to ESR1, and is also located on chromsone 13 similar to miR‐19a (Fig. 12). To investigate the biological effects of DLEU1 on ER expression, we first examined MCF‐7 and MDA‐MB‐231 cells. Figure 13A showed that the endogenous expression level of DLEU1 in MCF‐7 was higher than that in MDA‐MB‐231 cells (*P* < 0.05). Three siRNAs were used to knockdown DLEU1. The efficiency of the three siRNA candidates was confirmed by qRT‐PCR (Fig. 13B). Next, we used the most efficient sequence, siRNA 3, to transfect MCF‐7 cells. The ER expression was confirmed by western blot analysis (Fig. 13C), which showed that the knockdown of DLEU1 decreased the ER protein expression.

**Figure 14:**
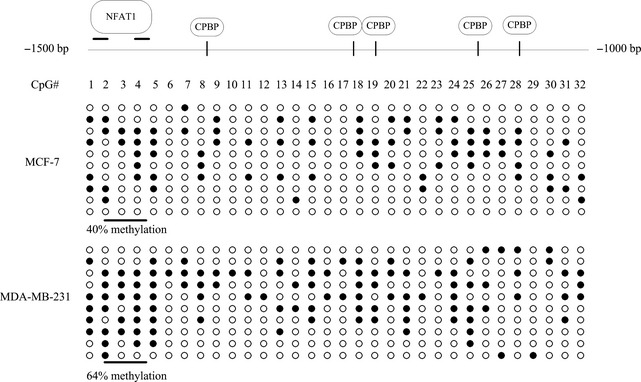
Bisulphite sequencing of lncRNA‐DLEU1 promoter region. The nucleotide sequence −1500 to −1000 bp from the transcription start site of the lncRNA‐DLEU1 promoter for the bisulphite sequencing analysis are shown. Genomic DNA from MCF‐7 and MDA‐MB‐231 cells was sodium bisulphite‐treated,PCR amplification and subcloned. The sequencing results from 10 clones were used for analysis. Each horizontal line represented the sequencing results of one subclone. Methylated CpG sites are shown as solid circles, whereas open circles indicated unmethylated CpG sites.

The lncRNA‐ DLEU1 maps to a critical region at chromosomal band 13q14.3 that is frequently deleted in solid tumours and haematopoietic malignancies, such as chronic lymphocytic leukaemia (CLL)^53^, ^54^. Although no point mutations have been found in the protein‐coding candidate genes at 13q14.3, these genes are deregulated in malignant cells, suggesting that epigenetic aberrations may play a major role in tumour suppressor mechanism 55. Because epigenetic aberrations have been found in the promoter region of lnc‐RNA DLEU1 in CLL cells 56, we investigated the epigenetic status of CpG islands lncRNA‐DLEU1 gene promoter region (−1500 to −1000) in MCF‐7 and MDA‐MB‐231 cells. The CpG‐rich region of the promoter upstream −1500 to −1000 bp (a total of 32 CpG sites) was sequenced after bisulphite modification of genomic DNA from MCF‐7 and MDA‐MB‐231cells. The methylation analysis indicated that the CpG sites examined were more methylated in MDA‐MB‐231 cells than that in MCF‐7 cells (Fig. 14). Epigenetic aberrations in lncRNA‐DLEU1 in MDA‐MB‐231 cells may be correlated with the status of ER. However, the relationship of lncRNA‐DLEU1 and ER need to be validated further. Figure 14 also showed the transcription factors and their binding sites in this region, such as nuclear factor of activated T cells 1 (NFAT1) and core promoter binding protein. More specifically, there was a significant difference in methylation at the transcription factor NFAT1 binding region between MCF‐7 and MDA‐MB‐231 cells. NFAT1 has been implicated in cell motility 56. But whether this influences the phenotype of BC cells remains to be elucidated.

Although lncRNA‐related researches have increased rapidly, only few have been well characterized. For now, there are some lncRNAs associated with BC (as shown in Table S11). As mentioned above lncRNAs, our data showed that the expression of lncRNA‐H19 and XIST in MCF‐7 cells was more than that in MDA‐MB‐231, and lncRNA‐UCA1 lower than that in MDA‐MB‐231. For example, the H19 gene can affect the phenotype of human breast epithelial cells. Overexpression of H19 resulted in an increase of anchorage‐independent growth in MDA‐MB‐231 BC cells and therefore H19 has been considered as and oncogenic RNA in BC cell lines 12. However, in other works, introduction of H19 into several tumour cell lines caused tumour suppressor effects 57. So it needs further verification. But, the above lncRNAs were not involved in our networks, which may be due to the characteristics of MCF‐7 and MDA‐MB‐231. We should compare the expression profiles of ncRNA and mRNA between the tumour cell lines and normal cell lines, such as MCF‐10A, in further studies.

Several studies have investigated microarray or sequencing data and identified single lncRNAs with diagnostic power or cancer progression properties 58, 59, 60, 61. A number of lncRNA have been identified to be associated with cancer‐related genes. lncRNA‐mRNA interaction is very similar to the miRNA regulation of mRNA. lncRNA can bind to some mRNAs and one mRNA can be targeted by multiple lncRNAs. Also lncRNA can interact with miRNA as a sponge 62. In a recent research, a novel mechanism of tumourigenesis involving lncRNAs, mRNAs and microRNAs was presented, which is that association of lncRNA‐DANCR with CTNNB1 gene blocked the repressing effect of miR‐214, miR‐320a and miR‐199a on CTNNB1 63. Therefore, RNA–RNA interaction may be another realm to post‐transcription regulation. *Silico* studies is very necessary for the integration of known RNA–RNA interaction. There is a need for comprehensive mapping of the RNA–RNA interaction. Our work only presented a framework of ncRNA–mRNA network in MCF‐7 and MDA‐MB‐231 cell lines, which needs more efforts to quantification and annotation for networks.

## Conclusion

We analysed the mRNA and ncRNA profile of MCF‐7 and MDA‐MB‐231 BC cell lines with different ER status to provide mRNA–ncRNA networking, and then presented preliminary experimental supports linking ncRNA to ER expression. Our results increase the understanding of ncRNA–mRNA networks in BC cell lines. First, miRNA–mRNA and lncRNA–mRNA relationships were analysed at the same time. Second, highly stringent selection procedures enable us to validate the relationship with the greatest correlation. But our limitation is that we have not performed the integrative analysis of the miRNA–lncRNA interaction. And because the function of only a few lncRNAs have been described, further studies should be conducted to expand these findings in additional cell lines and tissues samples.

## Conflicts of interests

The authors declare that they have no competing interests.

## Author contribution

QW conceived and designed the experiments. FJ, LS, JJC performed the experiments. QW and JJC analysed the data. FC, ZL and LL contributed reagents/materials/analysis tools. QW wrote the article. All the authors read and approved the final manuscript.

## Supporting information


**Figure S1** Hierarchical clustering analysis of miRNA differentially expressed between MCF‐7 and MDA‐MB‐ 231 cells.

**Figure S2** Profiles of lncRNAs and mRNAs in MCF‐7 and MDA‐MB‐ 231 cells.

**Figure S3** The overall structures of constructed co‐expression network of MCF‐7 cell.

**Figure S4** The overall structures of constructed co‐expression network of MDA‐MB‐231 cell.

**Figure S5** ESR1‐related co‐expression miRNA and lncRNA in MCF‐7 cells.

**Figure S6** ESR1‐related co‐expression miRNA and lncRNA in MDA‐MB‐231 cells.

**Table S1** Source of lncRNAs contained on Agilent human lncRNA microarray v.2.0.

**Table S2** The sequences of DELU1‐siRNA.

**Table S3** Highly expressed miRNAs in MCF‐7 cell.

**Table S4** Highly expressed miRNAs in MDA‐MB‐231 cell.

**Table S5** The details of the up‐regulated mRNAs in MCF‐7 cell compared with that in MDA‐MB‐231 cell.

**Table S6** The details of the up‐regulated lncRNAs in MCF‐7 cell compared with that in MDA‐MB‐231 cell.

**Table S7** The details of the down‐regulated lncRNAs in MCF‐7 cell compared with that in MDA‐MB‐231 cell.

**Table S8** The details of the down‐regulated mRNAs in MCF‐7 cell compared with that in MDA‐MB‐231 cell.

**Table S9** The candidate mRNAs for the co‐expression network.

**Table S10** The GO analysis of differential expressed gene in MCF‐7 *versus* MDA‐MB‐231 cell lines.

**Table S11** lncRNAs associated with breast cancer.
