# Supplementary figures and images for: Analysis of the miRNA–mRNA–lncRNA networks in ER+ and ER− breast cancer cell lines
Source: J Cell Mol Med. 2015 Sep 28;19(12):2874–87. doi: 10.1111/jcmm.12681 (PMC4687702; doi:10.1111/jcmm.12681)

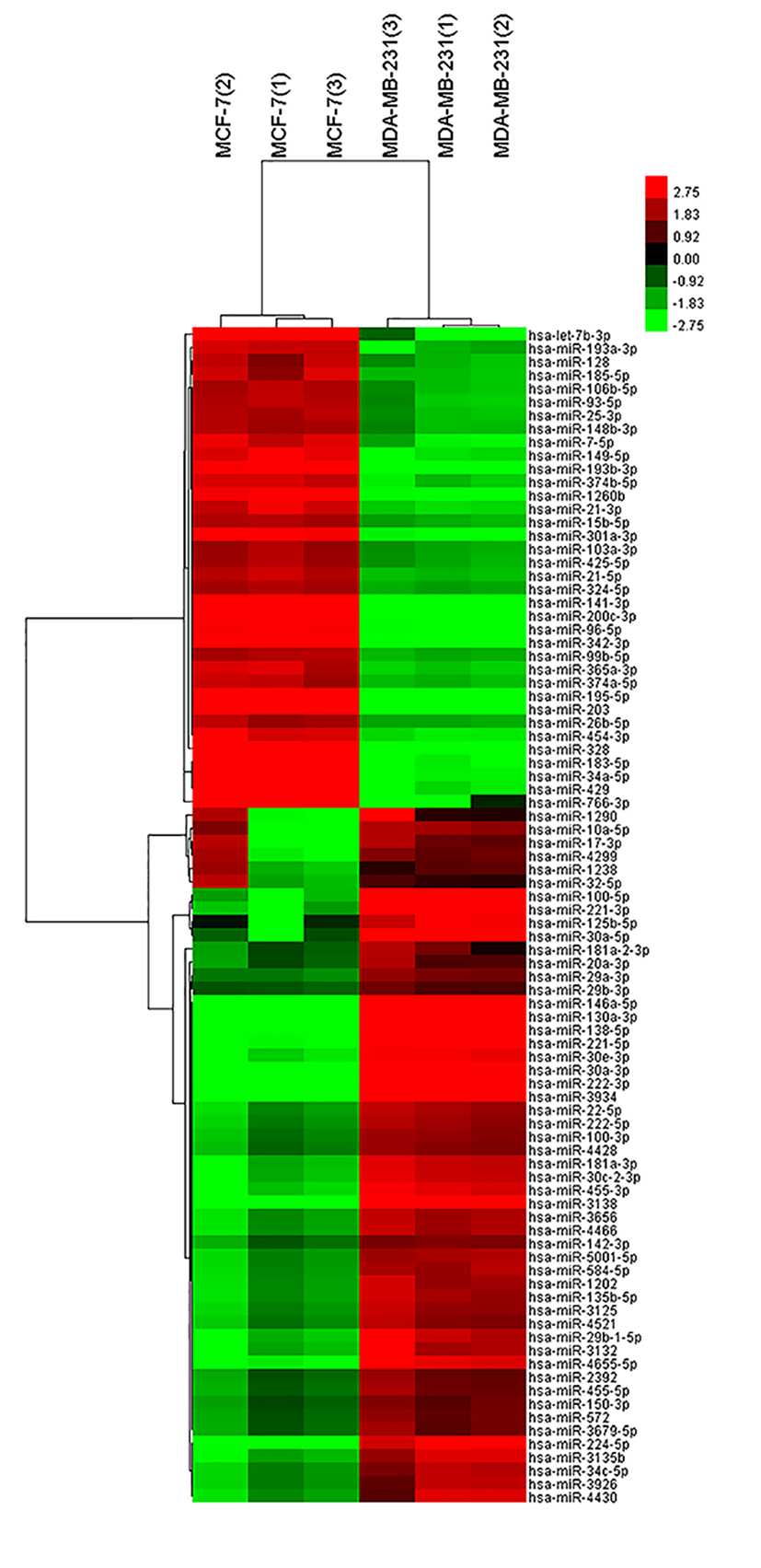

Supplement: Supplementary file 1 — Figure S1 Hierarchical clustering analysis of miRNA differentially expressed between MCF‐7 and MDA‐MB‐ 231 cells. [file JCMM-19-2874-s001.tif]

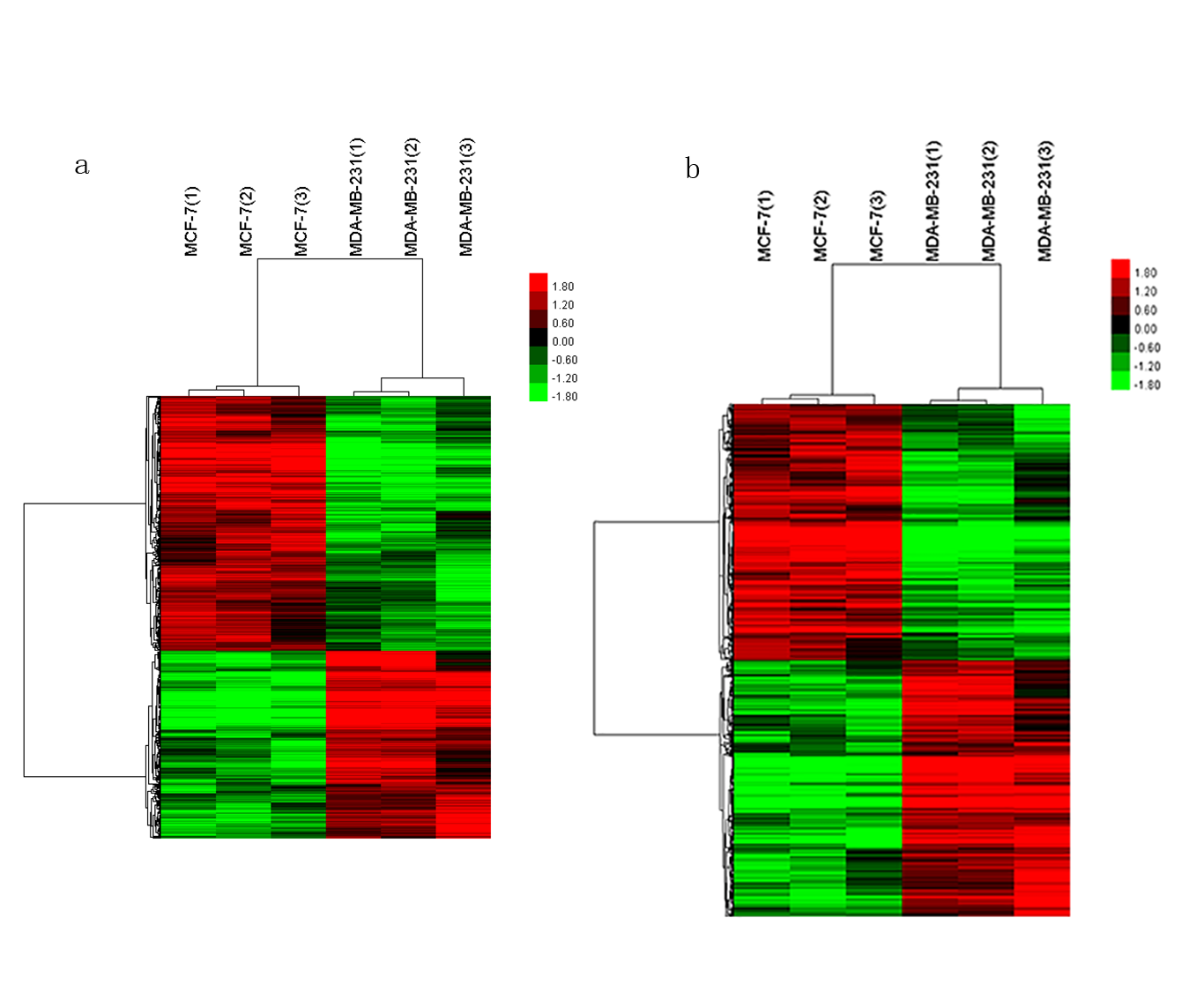

Supplement: Supplementary file 2 — Figure S2 Profiles of lncRNAs and mRNAs in MCF‐7 and MDA‐MB‐ 231 cells. [file JCMM-19-2874-s002.tif]
