# Supplementary material for: Analysis of the miRNA–mRNA–lncRNA networks in ER+ and ER− breast cancer cell lines
Source: J Cell Mol Med. 2015 Sep 28;19(12):2874–87. doi: 10.1111/jcmm.12681 (PMC4687702; doi:10.1111/jcmm.12681)
Supplement: Supplementary file 7 — Table S1 Source of lncRNAs contained on Agilent human lncRNA microarray v.2.0. [file JCMM-19-2874-s007.docx]

Table S1 Source of lncRNAs contained on Agilent human lncRNA microarray v2.0.

| Database | # of lncRNAs |
| --- | --- |
| imsRNA ( February 2011) | 848 |
| RefSeq ( February 2011) | 4,765 |
| UCSC ( February 2011) | 13,521 |
| ENSEMBL (November 2011) | 12,754 |
| lincRNA From GeneDev(November 2011) | 8,195 |
| Hox ncRNAs(April 2011) | 407 |
| lncRNAdb ( January 2011) | 78 |
| NRED( January 2011) | 1,289 |
| H-InvDB 7.5 | 17,203 |
| CombinedLitV2 | 529 |
| Antisense ncRNA pipeline ( January 2011) | 1,053 |
| snoRNA ( February 2011) | 389 |
| EvoFold( January 2011) | 47,109 |
| RNAzV2( January 2011) | 35,827 |
| Total (after removing redundant sequences) | 39,213 |
