# Supplementary material for: Analysis of the miRNA–mRNA–lncRNA networks in ER+ and ER− breast cancer cell lines
Source: J Cell Mol Med. 2015 Sep 28;19(12):2874–87. doi: 10.1111/jcmm.12681 (PMC4687702; doi:10.1111/jcmm.12681)
Supplement: Supplementary file 8 — Table S2 The sequences of DELU1‐siRNA. [file JCMM-19-2874-s008.doc]

**Table S2 The sequences of DELU1-siRNA**

| No. | siRNA1 | siRNA2 | siRNA3 |
| --- | --- | --- | --- |
| Target sequence | GCAGAAAGGAAGTTTACTT | GCATATACCAGAACTTCAT | GTAGCTATCCTAGTAGATA |
| Sense strand | 5‘ GCAGAAAGGAAGUUUACUU dTdT 3‘ | 5‘ GCAUAUACCAGAACUUCAU dTdT 3‘ | 5‘ GUAGCUAUCCUAGUAGAUA dTdT 3‘ |
| Anti-sense strand | 3‘ dTdT CGUCUUUCCUUCAAAUGAA 5‘ | 3‘ dTdT CGUAUAUGGUCUUGAAGUA 5‘ | 3‘ dTdT CAUCGAUAGGAUCAUCUAU 5‘ |
