# Supplementary material for: Analysis of the miRNA–mRNA–lncRNA networks in ER+ and ER− breast cancer cell lines
Source: J Cell Mol Med. 2015 Sep 28;19(12):2874–87. doi: 10.1111/jcmm.12681 (PMC4687702; doi:10.1111/jcmm.12681)
Supplement: Supplementary file 9 — Table S3 Highly expressed miRNAs in MCF‐7 cell. [file JCMM-19-2874-s009.doc]

Table S3 Highly expressed miRNAs in MCF-7 compared with MDA-MB-231 cells

| **Systematic_name** | **FC** |
| --- | --- |
| hsa-let-7b-3p | 44.77734 |
| hsa-miR-103a-3p | 2.05604 |
| hsa-miR-106b-5p | 2.578975 |
| hsa-miR-1260b | 13.2068 |
| hsa-miR-128 | 2.414402 |
| hsa-miR-141-3p | 10771.61 |
| hsa-miR-148b-3p | 2.297807 |
| hsa-miR-149-5p | 6.820013 |
| hsa-miR-15b-5p | 2.452519 |
| hsa-miR-183-5p | 77.89976 |
| hsa-miR-185-5p | 3.193911 |
| hsa-miR-193a-3p | 3.788077 |
| hsa-miR-193b-3p | 11.0053 |
| hsa-miR-195-5p | 537.6375 |
| hsa-miR-200c-3p | 7289.557 |
| hsa-miR-203 | 460.1832 |
| hsa-miR-21-3p | 4.568306 |
| hsa-miR-21-5p | 3.087831 |
| hsa-miR-25-3p | 2.607036 |
| hsa-miR-26b-5p | 2.299722 |
| hsa-miR-301a-3p | 9.692651 |
| hsa-miR-324-5p | 2.280983 |
| hsa-miR-328 | 115.1741 |
| hsa-miR-342-3p | 35.29468 |
| hsa-miR-34a-5p | 143.4939 |
| hsa-miR-365a-3p | 3.935787 |
| hsa-miR-374a-5p | 2.895986 |
| hsa-miR-374b-5p | 4.222325 |
| hsa-miR-425-5p | 2.192843 |
| hsa-miR-429 | 101.1913 |
| hsa-miR-454-3p | 5.678928 |
| hsa-miR-7-5p | 4.806823 |
| hsa-miR-766-3p | 26.95579 |
| hsa-miR-93-5p | 2.809985 |
| hsa-miR-96-5p | 8.475113 |
| hsa-miR-99b-5p | 2.554621 |

The highlighted miRNAs are that only detected in MCF-7. FC: fold change.
