# Supplementary material for: Analysis of the miRNA–mRNA–lncRNA networks in ER+ and ER− breast cancer cell lines
Source: J Cell Mol Med. 2015 Sep 28;19(12):2874–87. doi: 10.1111/jcmm.12681 (PMC4687702; doi:10.1111/jcmm.12681)
Supplement: Supplementary file 10 — Table S4 Highly expressed miRNAs in MDA‐MB‐231 cell. [file JCMM-19-2874-s010.docx]

**Table S4 Highly expressed miRNAs in MDA-MB-231 compared with MCF-7 cells**

| **Systematic_name** | **FC (abs)** |
| --- | --- |
| hsa-miR-100-3p | 49.54599 |
| hsa-miR-100-5p | 785.5418 |
| hsa-miR-10a-5p | 60.91945 |
| hsa-miR-1202 | 74.86335 |
| hsa-miR-1238 | 17.00293 |
| hsa-miR-125b-5p | 188.9742 |
| hsa-miR-1290 | 31.2054 |
| hsa-miR-130a-3p | 4040.39 |
| hsa-miR-135b-5p | 76.41087 |
| hsa-miR-138-5p | 486.863 |
| hsa-miR-142-3p | 36.36084 |
| hsa-miR-146a-5p | 2593.665 |
| hsa-miR-150-3p | 28.5184 |
| hsa-miR-17-3p | 37.18408 |
| hsa-miR-17-5p | 2.441438 |
| hsa-miR-181a-2-3p | 27.56104 |
| hsa-miR-181a-3p | 117.1242 |
| hsa-miR-181a-5p | 3.415843 |
| hsa-miR-19a-3p | 2.618535 |
| hsa-miR-19b-3p | 2.859846 |
| hsa-miR-20a-3p | 29.43085 |
| hsa-miR-20a-5p | 2.577029 |
| hsa-miR-20b-5p | 3.65593 |
| hsa-miR-22-5p | 72.90011 |
| hsa-miR-221-3p | 738.911 |
| hsa-miR-221-5p | 402.9037 |
| hsa-miR-222-3p | 1537.452 |
| hsa-miR-222-5p | 61.69789 |
| hsa-miR-224-5p | 618.1064 |
| hsa-miR-2392 | 32.71846 |
| hsa-miR-23a-3p | 2.679593 |
| hsa-miR-29a-3p | 36.70899 |
| hsa-miR-29b-1-5p | 140.6322 |
| hsa-miR-29b-3p | 21.01913 |
| hsa-miR-29c-3p | 3.036827 |
| hsa-miR-30a-3p | 795.9919 |
| hsa-miR-30a-5p | 292.7333 |
| hsa-miR-30c-2-3p | 128.623 |
| hsa-miR-30c-5p | 4.706453 |
| hsa-miR-30e-3p | 214.9479 |
| hsa-miR-3125 | 65.00286 |
| hsa-miR-3132 | 114.6266 |
| hsa-miR-3135b | 108.6 |
| hsa-miR-3138 | 461.458 |
| hsa-miR-32-5p | 12.76253 |
| hsa-miR-34c-5p | 64.51563 |
| hsa-miR-3656 | 76.40021 |
| hsa-miR-3679-5p | 34.68006 |
| hsa-miR-3926 | 62.02774 |
| hsa-miR-3934 | 885.5583 |
| hsa-miR-424-5p | 3.67931 |
| hsa-miR-4284 | 2.458896 |
| hsa-miR-4299 | 29.36091 |
| hsa-miR-4428 | 43.94997 |
| hsa-miR-4430 | 78.53487 |
| hsa-miR-4443 | 3.501015 |
| hsa-miR-4466 | 78.82107 |
| hsa-miR-4521 | 53.30149 |
| hsa-miR-455-3p | 173.0622 |
| hsa-miR-455-5p | 36.84429 |
| hsa-miR-4655-5p | 335.9972 |
| hsa-miR-5001-5p | 66.75767 |
| hsa-miR-572 | 32.39946 |
| hsa-miR-584-5p | 68.22371 |
| hsa-miR-92a-3p | 3.352826 |

The highlighted miRNAs are that only detected in MDA-MB-231. FC: fold change.
