# Supplementary material for: Analysis of the miRNA–mRNA–lncRNA networks in ER+ and ER− breast cancer cell lines
Source: J Cell Mol Med. 2015 Sep 28;19(12):2874–87. doi: 10.1111/jcmm.12681 (PMC4687702; doi:10.1111/jcmm.12681)
Supplement: Supplementary file 16 — Table S10 The GO analysis of differential expressed gene in MCF‐7 versus MDA‐MB‐231 cell lines. [file JCMM-19-2874-s016.doc]

**Table S10 GO analysis of differential expressed gene in MCF-7 vs. MDA-MB-231 cell lines**

| **go_id** | **go_name** | **go_diffgene_count** | **pvalue** | **FDR** |
| --- | --- | --- | --- | --- |
| GO:0007165 | signal transduction | 35 | 1.40E-10 | 1.97E-08 |
| GO:0006915 | apoptotic process | 22 | 6.19E-07 | 1.34E-05 |
| GO:0007173 | epidermal growth factor receptor signaling pathway | 11 | 3.06E-06 | 5.22E-05 |
| GO:0016477 | cell migration | 9 | 3.82E-06 | 6.30E-05 |
| GO:0043066 | negative regulation of apoptotic process | 17 | 7.77E-06 | 0.000112636 |
| GO:0045787 | positive regulation of cell cycle | 5 | 1.13E-05 | 0.000147965 |
| GO:0060749 | mammary gland alveolus development | 4 | 1.87E-05 | 0.000218936 |
| GO:0008286 | insulin receptor signaling pathway | 9 | 2.28E-05 | 0.000256171 |
| GO:0008284 | positive regulation of cell proliferation | 14 | 7.68E-05 | 0.000651709 |
| GO:0035556 | intracellular signal transduction | 11 | 0.000167385 | 0.001205807 |
| GO:0055085 | transmembrane transport | 15 | 0.000380664 | 0.002129912 |
| GO:0007155 | cell adhesion | 13 | 0.000781086 | 0.003201141 |
| GO:0008285 | negative regulation of cell proliferation | 11 | 0.001211835 | 0.004172518 |
| GO:0030335 | positive regulation of cell migration | 6 | 0.001448214 | 0.004729844 |
| GO:0042493 | response to drug | 9 | 0.002355281 | 0.006423967 |
| GO:0001525 | angiogenesis | 7 | 0.005880697 | 0.010776141 |
| GO:0045087 | innate immune response | 12 | 0.012662316 | 0.015098304 |
| GO:0007165 | signal transduction | 57 | 3.18E-23 | 7.50E-21 |
| GO:0008284 | positive regulation of cell proliferation | 28 | 5.28E-14 | 3.12E-12 |
| GO:0007173 | epidermal growth factor receptor signaling pathway | 19 | 5.93E-13 | 2.33E-11 |
| GO:0043066 | negative regulation of apoptotic process | 27 | 1.66E-11 | 4.35E-10 |
| GO:0007155 | cell adhesion | 25 | 1.31E-10 | 2.80E-09 |
| GO:0008283 | cell proliferation | 21 | 4.55E-10 | 8.56E-09 |
| GO:0030335 | positive regulation of cell migration | 13 | 9.17E-10 | 1.55E-08 |
| GO:0001525 | angiogenesis | 15 | 1.62E-08 | 2.11E-07 |
| GO:0007179 | transforming growth factor beta receptor signaling pathway | 12 | 2.14E-08 | 2.66E-07 |
| GO:0030198 | extracellular matrix organization | 15 | 2.93E-08 | 3.38E-07 |
| GO:0016477 | cell migration | 11 | 1.83E-07 | 1.62E-06 |
| GO:0006915 | apoptotic process | 25 | 1.96E-07 | 1.72E-06 |
| GO:0048010 | vascular endothelial growth factor receptor signaling pathway | 6 | 5.06E-07 | 3.80E-06 |
| GO:0008360 | regulation of cell shape | 10 | 5.81E-07 | 4.21E-06 |
| GO:0045087 | innate immune response | 22 | 6.09E-07 | 4.36E-06 |
| GO:0045766 | positive regulation of angiogenesis | 9 | 7.69E-07 | 5.25E-06 |
| GO:0008286 | insulin receptor signaling pathway | 11 | 1.66E-06 | 9.73E-06 |
| GO:0001837 | epithelial to mesenchymal transition | 6 | 1.99E-06 | 1.12E-05 |
| GO:0002053 | positive regulation of mesenchymal cell proliferation | 6 | 2.94E-06 | 1.54E-05 |
| GO:0007050 | cell cycle arrest | 10 | 3.34E-06 | 1.69E-05 |
| GO:0000902 | cell morphogenesis | 6 | 5.06E-05 | 0.00017653 |
| GO:0042127 | regulation of cell proliferation | 8 | 5.40E-05 | 0.00018648 |
| GO:0006917 | induction of apoptosis | 10 | 7.43E-05 | 0.00024055 |
| GO:0008285 | negative regulation of cell proliferation | 14 | 0.000101431 | 0.00030475 |
| GO:0006954 | inflammatory response | 12 | 0.000238609 | 0.00055219 |
| GO:0042493 | response to drug | 10 | 0.001989884 | 0.00246483 |
| GO:0043065 | positive regulation of apoptotic process | 8 | 0.003213668 | 0.00336435 |
| GO:0007049 | cell cycle | 8 | 0.00708808 | 0.00575705 |
| GO:0006955 | immune response | 10 | 0.011746788 | 0.0075885 |

Highlighted represents GO term that downreglated genes are involved in. FDR: false discovery rate
