# Supplementary material for: Analysis of the miRNA–mRNA–lncRNA networks in ER+ and ER− breast cancer cell lines
Source: J Cell Mol Med. 2015 Sep 28;19(12):2874–87. doi: 10.1111/jcmm.12681 (PMC4687702; doi:10.1111/jcmm.12681)
Supplement: Supplementary file 17 — Table S11 lncRNAs associated with breast cancer. [file JCMM-19-2874-s017.doc]

Table S11 lncRNAs associated with breast cancer

| lncRNAs | ID | Reference* |
| --- | --- | --- |
| BCAR4 | ENSG00000262117 | Meijer D T et al. (2006)  Angulo L et al. (2013) |
| UCA1/CUDR/LINC00178 | ENSG00000214049 | Tsang W P et al. (2007)  Zhang Z et al. (2012) |
| SRA/SRA-1/STRAA1 | ENSG00000213523 | [Lanz R B](http://www.ncbi.nlm.nih.gov/pubmed?term=Lanz RB%5BAuthor%5D&cauthor=true&cauthor_uid=10199399) et al. (1999)  Cooper C et al. (2009) |
| LSINCT5 | HGNC:37824 | Silva J M et al. (2011) |
| BC200/BCYRN1/LINC00004/NCRNA00004 | ENSG00000236824 | Iacoangeli A et al. (2004) |
| HOTAIR/HOXC-AS4/HOXC11-AS1/NCRNA00072 | **ENSG00000228630** | [**Gupta R A**](http://www.ncbi.nlm.nih.gov/pubmed?term=Gupta RA%5BAuthor%5D&cauthor=true&cauthor_uid=20393566) et al. (2010) |
| GAS5/SNHG2/NCRNA00030 | **ENSG00000234741** | [**Mourtada-Maarabouni M**](http://www.ncbi.nlm.nih.gov/pubmed?term=Mourtada-Maarabouni M%5BAuthor%5D&cauthor=true&cauthor_uid=18836484) et al. (2009) |
| MALAT-1/NEAT2 /HCN/LINC00047/NCRNA00047/PRO1073 | ENSG00000251562 | Tani, H et al. (2010)  Ji P et al. (2003)  Kryger R et al. (2012)  Lai M C et al. (2012)  Xu C et al. (2011) |
| H19/ASM/ASM1/D11S813E/LINC00008/NCRNA00008 | ENSG00000130600 | Looijenga L H et al. (1997)  Lottin S et al. (2002)  Berteaux N et al. (2008) |
| Zfas1/ZNFX1-AS1/NCRNA00275/HSUP1/HSUP2 | ENSG00000177410 | Shore A N et al. (2012) |
| LOC554202/MIR31HG | ENSG00000171889 | Augoff K et al. (2012) |

*References:

1. Meijer, D., T. van Agthoven, P. T. Bosma, K. Nooter and L. C. Dorssers (2006). "Functional screen for genes responsible for tamoxifen resistance in human breast cancer cells." Mol Cancer Res 4(6): 379-386.
2. Angulo, L.[Perreau C](http://www.ncbi.nlm.nih.gov/pubmed/?term=Perreau C%5BAuthor%5D&cauthor=true&cauthor_uid=23202989), [Lakhdari N](http://www.ncbi.nlm.nih.gov/pubmed/?term=Lakhdari N%5BAuthor%5D&cauthor=true&cauthor_uid=23202989), [Uzbekov R](http://www.ncbi.nlm.nih.gov/pubmed/?term=Uzbekov R%5BAuthor%5D&cauthor=true&cauthor_uid=23202989), [Papillier P](http://www.ncbi.nlm.nih.gov/pubmed/?term=Papillier P%5BAuthor%5D&cauthor=true&cauthor_uid=23202989), [Freret S](http://www.ncbi.nlm.nih.gov/pubmed/?term=Freret S%5BAuthor%5D&cauthor=true&cauthor_uid=23202989), [Cadoret V](http://www.ncbi.nlm.nih.gov/pubmed/?term=Cadoret V%5BAuthor%5D&cauthor=true&cauthor_uid=23202989), [Guyader-Joly C](http://www.ncbi.nlm.nih.gov/pubmed/?term=Guyader-Joly C%5BAuthor%5D&cauthor=true&cauthor_uid=23202989), [Royere D](http://www.ncbi.nlm.nih.gov/pubmed/?term=Royere D%5BAuthor%5D&cauthor=true&cauthor_uid=23202989), [Ponsart C](http://www.ncbi.nlm.nih.gov/pubmed/?term=Ponsart C%5BAuthor%5D&cauthor=true&cauthor_uid=23202989), [Uzbekova S](http://www.ncbi.nlm.nih.gov/pubmed/?term=Uzbekova S%5BAuthor%5D&cauthor=true&cauthor_uid=23202989), [Dalbies-Tran R](http://www.ncbi.nlm.nih.gov/pubmed/?term=Dalbies-Tran R%5BAuthor%5D&cauthor=true&cauthor_uid=23202989). (2013). Breast-cancer anti-estrogen resistance 4 (BCAR4) encodes a novel maternal-effect protein in bovine and is expressed in the oocyte of humans and other non-rodent mammals. Hum Reprod 28(2): 430-441.
3. Tsang, W. P., T. W. Wong, A. H. Cheung, C. N. Co and T. T. Kwok (2007). Induction of drug resistance and transformation in human cancer cells by the noncoding RNA CUDR. RNA 13(6): 890-898.
4. Zhang, Z., [Hao H](http://www.ncbi.nlm.nih.gov/pubmed/?term=Hao H%5BAuthor%5D&cauthor=true&cauthor_uid=22490897), [Zhang CJ](http://www.ncbi.nlm.nih.gov/pubmed/?term=Zhang CJ%5BAuthor%5D&cauthor=true&cauthor_uid=22490897), [Yang XY](http://www.ncbi.nlm.nih.gov/pubmed/?term=Yang XY%5BAuthor%5D&cauthor=true&cauthor_uid=22490897), [He Q](http://www.ncbi.nlm.nih.gov/pubmed/?term=He Q%5BAuthor%5D&cauthor=true&cauthor_uid=22490897), [Lin J](http://www.ncbi.nlm.nih.gov/pubmed/?term=Lin J%5BAuthor%5D&cauthor=true&cauthor_uid=22490897) (2012).Evaluation of novel gene UCA1 as a tumor biomarker for the detection of bladder cancer. Zhonghua Yi Xue Za Zhi 92(6): 384-387.
5. [Lanz RB](http://www.ncbi.nlm.nih.gov/pubmed?term=Lanz RB%5BAuthor%5D&cauthor=true&cauthor_uid=10199399), [McKenna NJ](http://www.ncbi.nlm.nih.gov/pubmed?term=McKenna NJ%5BAuthor%5D&cauthor=true&cauthor_uid=10199399), [Onate SA](http://www.ncbi.nlm.nih.gov/pubmed?term=Onate SA%5BAuthor%5D&cauthor=true&cauthor_uid=10199399), [Albrecht U](http://www.ncbi.nlm.nih.gov/pubmed?term=Albrecht U%5BAuthor%5D&cauthor=true&cauthor_uid=10199399), [Wong J](http://www.ncbi.nlm.nih.gov/pubmed?term=Wong J%5BAuthor%5D&cauthor=true&cauthor_uid=10199399), [Tsai SY](http://www.ncbi.nlm.nih.gov/pubmed?term=Tsai SY%5BAuthor%5D&cauthor=true&cauthor_uid=10199399), [Tsai MJ](http://www.ncbi.nlm.nih.gov/pubmed?term=Tsai MJ%5BAuthor%5D&cauthor=true&cauthor_uid=10199399), [O'Malley BW](http://www.ncbi.nlm.nih.gov/pubmed?term=O'Malley BW%5BAuthor%5D&cauthor=true&cauthor_uid=10199399)(1999). A steroid receptor coactivator, SRA, functions as an RNA and is present in an SRC-1 complex. [Cell.](http://www.ncbi.nlm.nih.gov/pubmed/10199399#%23) 97(1):17-27.
6. Cooper, C., [Guo J](http://www.ncbi.nlm.nih.gov/pubmed/?term=Guo J%5BAuthor%5D&cauthor=true&cauthor_uid=19483093), [Yan Y](http://www.ncbi.nlm.nih.gov/pubmed/?term=Yan Y%5BAuthor%5D&cauthor=true&cauthor_uid=19483093), [Chooniedass-Kothari S](http://www.ncbi.nlm.nih.gov/pubmed/?term=Chooniedass-Kothari S%5BAuthor%5D&cauthor=true&cauthor_uid=19483093), [Hube F](http://www.ncbi.nlm.nih.gov/pubmed/?term=Hube F%5BAuthor%5D&cauthor=true&cauthor_uid=19483093), [Hamedani MK](http://www.ncbi.nlm.nih.gov/pubmed/?term=Hamedani MK%5BAuthor%5D&cauthor=true&cauthor_uid=19483093), [Murphy LC](http://www.ncbi.nlm.nih.gov/pubmed/?term=Murphy LC%5BAuthor%5D&cauthor=true&cauthor_uid=19483093), [Myal Y](http://www.ncbi.nlm.nih.gov/pubmed/?term=Myal Y%5BAuthor%5D&cauthor=true&cauthor_uid=19483093), [Leygue E](http://www.ncbi.nlm.nih.gov/pubmed/?term=Leygue E%5BAuthor%5D&cauthor=true&cauthor_uid=19483093). (2009). Increasing the relative expression of endogenous non-coding Steroid Receptor RNA Activator (SRA) in human breast cancer cells using modified oligonucleotides. Nucleic Acids Res 37(13): 4518-4531.
7. Silva, J. M., N. J. Boczek, M. W. Berres, X. Ma and D. I. Smith. (2011). LSINCT5 is over expressed in breast and ovarian cancer and affects cellular proliferation. RNA Biol 8(3): 496-505.
8. Iacoangeli, A., Y. Lin, E. J. Morley, I. A. Muslimov, R. Bianchi, J. Reilly, J. Weedon, R. Diallo, W. Bocker and H. Tiedge.(2004). BC200 RNA in invasive and preinvasive breast cancer. Carcinogenesis 25(11): 2125-2133.
9. [Gupta RA](http://www.ncbi.nlm.nih.gov/pubmed?term=Gupta RA%5BAuthor%5D&cauthor=true&cauthor_uid=20393566), [Shah N](http://www.ncbi.nlm.nih.gov/pubmed?term=Shah N%5BAuthor%5D&cauthor=true&cauthor_uid=20393566), [Wang KC](http://www.ncbi.nlm.nih.gov/pubmed?term=Wang KC%5BAuthor%5D&cauthor=true&cauthor_uid=20393566), [Kim J](http://www.ncbi.nlm.nih.gov/pubmed?term=Kim J%5BAuthor%5D&cauthor=true&cauthor_uid=20393566), [Horlings HM](http://www.ncbi.nlm.nih.gov/pubmed?term=Horlings HM%5BAuthor%5D&cauthor=true&cauthor_uid=20393566), [Wong DJ](http://www.ncbi.nlm.nih.gov/pubmed?term=Wong DJ%5BAuthor%5D&cauthor=true&cauthor_uid=20393566), [Tsai MC](http://www.ncbi.nlm.nih.gov/pubmed?term=Tsai MC%5BAuthor%5D&cauthor=true&cauthor_uid=20393566), [Hung T](http://www.ncbi.nlm.nih.gov/pubmed?term=Hung T%5BAuthor%5D&cauthor=true&cauthor_uid=20393566), [Argani P](http://www.ncbi.nlm.nih.gov/pubmed?term=Argani P%5BAuthor%5D&cauthor=true&cauthor_uid=20393566), [Rinn JL](http://www.ncbi.nlm.nih.gov/pubmed?term=Rinn JL%5BAuthor%5D&cauthor=true&cauthor_uid=20393566), [Wang Y](http://www.ncbi.nlm.nih.gov/pubmed?term=Wang Y%5BAuthor%5D&cauthor=true&cauthor_uid=20393566), [Brzoska P](http://www.ncbi.nlm.nih.gov/pubmed?term=Brzoska P%5BAuthor%5D&cauthor=true&cauthor_uid=20393566), [Kong B](http://www.ncbi.nlm.nih.gov/pubmed?term=Kong B%5BAuthor%5D&cauthor=true&cauthor_uid=20393566), [Li R](http://www.ncbi.nlm.nih.gov/pubmed?term=Li R%5BAuthor%5D&cauthor=true&cauthor_uid=20393566), [West RB](http://www.ncbi.nlm.nih.gov/pubmed?term=West RB%5BAuthor%5D&cauthor=true&cauthor_uid=20393566), [van de Vijver MJ](http://www.ncbi.nlm.nih.gov/pubmed?term=van de Vijver MJ%5BAuthor%5D&cauthor=true&cauthor_uid=20393566), [Sukumar S](http://www.ncbi.nlm.nih.gov/pubmed?term=Sukumar S%5BAuthor%5D&cauthor=true&cauthor_uid=20393566), [Chang HY](http://www.ncbi.nlm.nih.gov/pubmed?term=Chang HY%5BAuthor%5D&cauthor=true&cauthor_uid=20393566)(2010). Long non-coding RNA HOTAIR reprograms chromatin state to promote cancer metastasis. [Nature.](http://www.ncbi.nlm.nih.gov/pubmed/?term=Long+non-coding+RNA+HOTAIR+reprograms+chromatin+state+to+promote+cancer+metastasis．#%23) 464(7291):1071-6.
10. [Mourtada-Maarabouni M](http://www.ncbi.nlm.nih.gov/pubmed?term=Mourtada-Maarabouni M%5BAuthor%5D&cauthor=true&cauthor_uid=18836484), [Pickard MR](http://www.ncbi.nlm.nih.gov/pubmed?term=Pickard MR%5BAuthor%5D&cauthor=true&cauthor_uid=18836484), [Hedge VL](http://www.ncbi.nlm.nih.gov/pubmed?term=Hedge VL%5BAuthor%5D&cauthor=true&cauthor_uid=18836484), [Farzaneh F](http://www.ncbi.nlm.nih.gov/pubmed?term=Farzaneh F%5BAuthor%5D&cauthor=true&cauthor_uid=18836484), [Williams GT](http://www.ncbi.nlm.nih.gov/pubmed?term=Williams GT%5BAuthor%5D&cauthor=true&cauthor_uid=18836484).(2009).GAS5, a non-protein-coding RNA, controls apoptosis and is downregulated in breast cancer. Oncogene 28(2):195-208.
11. Tani, H., [Nakamura Y](http://www.ncbi.nlm.nih.gov/pubmed/?term=Nakamura Y%5BAuthor%5D&cauthor=true&cauthor_uid=22491206), [Ijiri K](http://www.ncbi.nlm.nih.gov/pubmed/?term=Ijiri K%5BAuthor%5D&cauthor=true&cauthor_uid=22491206), [Akimitsu N](http://www.ncbi.nlm.nih.gov/pubmed/?term=Akimitsu N%5BAuthor%5D&cauthor=true&cauthor_uid=22491206). (2010). Stability of MALAT-1, a nuclear long non-coding RNA in mammalian cells, varies in various cancer cells. Drug Discov Ther 4(4): 235-239.
12. Ji, P., S. Diederichs, W. Wang, S. Boing, R. Metzger, P. M. Schneider, N. Tidow, B. Brandt, H. Buerger, E. Bulk, M. Thomas, W. E. Berdel, H. Serve and C. Muller-Tidow (2003).MALAT-1, a novel noncoding RNA, and thymosin beta4 predict metastasis and survival in early-stage non-small cell lung cancer. Oncogene 22(39): 8031-8041.
13. Kryger, R., L. Fan, P. A. Wilce and V. Jaquet (2012). MALAT-1, a non protein-coding RNA is upregulated in the cerebellum, hippocampus and brain stem of human alcoholics. Alcohol 46(7): 629-634.
14. Lai, M. C., Z. Yang, L. Zhou, Q. Q. Zhu, H. Y. Xie, F. Zhang, L. M. Wu, L. M. Chen and S. S. Zheng (2012). Long non-coding RNA MALAT-1 overexpression predicts tumor recurrence of hepatocellular carcinoma after liver transplantation. Med Oncol 29(3): 1810-1816.
15. Xu, C., M. Yang, J. Tian, X. Wang and Z. Li (2011). MALAT-1: a long non-coding RNA and its important 3' end functional motif in colorectal cancer metastasis. Int J Oncol 39(1): 169-175.
16. Looijenga, L. H., A. J. Verkerk, N. De Groot, A. A. Hochberg and J. W. Oosterhuis (1997). "H19 in normal development and neoplasia." Mol Reprod Dev 46(3): 419-439.
17. Lottin, S., E. Adriaenssens, T. Dupressoir, N. Berteaux, C. Montpellier, J. Coll, T. Dugimont and J. J. Curgy (2002). Overexpression of an ectopic H19 gene enhances the tumorigenic properties of breast cancer cells. Carcinogenesis 23(11): 1885-1895
18. Berteaux, N., [Aptel N](http://www.ncbi.nlm.nih.gov/pubmed/?term=Aptel N%5BAuthor%5D&cauthor=true&cauthor_uid=18794369), [Cathala G](http://www.ncbi.nlm.nih.gov/pubmed/?term=Cathala G%5BAuthor%5D&cauthor=true&cauthor_uid=18794369), [Genton C](http://www.ncbi.nlm.nih.gov/pubmed/?term=Genton C%5BAuthor%5D&cauthor=true&cauthor_uid=18794369), [Coll J](http://www.ncbi.nlm.nih.gov/pubmed/?term=Coll J%5BAuthor%5D&cauthor=true&cauthor_uid=18794369), [Daccache A](http://www.ncbi.nlm.nih.gov/pubmed/?term=Daccache A%5BAuthor%5D&cauthor=true&cauthor_uid=18794369), [Spruyt N](http://www.ncbi.nlm.nih.gov/pubmed/?term=Spruyt N%5BAuthor%5D&cauthor=true&cauthor_uid=18794369), [Hondermarck H](http://www.ncbi.nlm.nih.gov/pubmed/?term=Hondermarck H%5BAuthor%5D&cauthor=true&cauthor_uid=18794369), [Dugimont T](http://www.ncbi.nlm.nih.gov/pubmed/?term=Dugimont T%5BAuthor%5D&cauthor=true&cauthor_uid=18794369), [Curgy JJ](http://www.ncbi.nlm.nih.gov/pubmed/?term=Curgy JJ%5BAuthor%5D&cauthor=true&cauthor_uid=18794369), [Forné T](http://www.ncbi.nlm.nih.gov/pubmed/?term=Forné T%5BAuthor%5D&cauthor=true&cauthor_uid=18794369), [Adriaenssens E](http://www.ncbi.nlm.nih.gov/pubmed/?term=Adriaenssens E%5BAuthor%5D&cauthor=true&cauthor_uid=18794369). (2008). A novel H19 antisense RNA overexpressed in breast cancer contributes to paternal IGF2 expression. Mol Cell Biol 28(22): 6731-6745.
19. Shore, A. N., J. I. Herschkowitz and J. M. Rosen (2012). Noncoding RNAs involved in mammary gland development and tumorigenesis: there's a long way to go. J Mammary Gland Biol Neoplasia 17(1): 43-58
20. Augoff, K., B. McCue, E. F. Plow and K. Sossey-Alaoui (2012). miR-31 and its host gene lncRNA LOC554202 are regulated by promoter hypermethylation in triple-negative breast cancer. Mol Cancer 11: 5.
